# Osteoblast-Specific Transcription Factor Osterix Increases Vitamin D Receptor Gene Expression in Osteoblasts

**DOI:** 10.1371/journal.pone.0026504

**Published:** 2011-10-18

**Authors:** Chi Zhang, Wanjin Tang, Yang Li, Fan Yang, Diane R. Dowd, Paul N. MacDonald

**Affiliations:** 1 Bone Research Laboratory, Texas Scottish Rite Hospital for Children, University of Texas Southwestern Medical Center at Dallas, Dallas, Texas, United States of America; 2 Department of Orthopedic Surgery, University of Texas Southwestern Medical Center at Dallas, Dallas, Texas, United States of America; 3 Department of Pharmacology, University of Texas Southwestern Medical Center at Dallas, Dallas, Texas, United States of America; 4 Department of Pharmacology, Case Western Reserve University, Cleveland, Ohio, United States of America; INSERM, UMR-S747, France

## Abstract

Osterix (Osx) is an osteoblast-specific transcription factor required for osteoblast differentiation from mesenchymal stem cells. In *Osx* knock-out mice, no bone formation occurs. The vitamin D receptor (VDR) is a member of the nuclear hormone receptor superfamily that regulates target gene transcription to ensure appropriate control of calcium homeostasis and bone development. Here, we provide several lines of evidence that show that the VDR gene is a target for transcriptional regulation by Osx in osteoblasts. For example, calvaria obtained from Osx-null embryos displayed dramatic reductions in VDR expression compared to wild-type calvaria. Stable overexpression of Osx stimulated VDR expression in C2C12 mesenchymal cells. Inhibition of Osx expression by siRNA led to downregulation of VDR. In contrast, Osx levels remained unchanged in osteoblasts in VDR-null mice. Mechanistic approaches using transient transfection assays showed that Osx directly activated a 1 kb fragment of the *VDR* promoter in a dose-dependent manner. To define the region of the *VDR* promoter that was responsive to Osx, a series of VDR promoter deletion mutants were examined and the minimal Osx-responsive region was refined to the proximal 120 bp of the *VDR* promoter. Additional point mutants were used to identify two GC-rich regions that were responsible for *VDR* promoter activation by Osx. Chromatin immunoprecipitation assays demonstrated that endogenous Osx was associated with the native *VDR* promoter in primary osteoblasts *in vivo*. Cumulatively, these data strongly support a direct regulatory role for Osx in VDR gene expression. They further provide new insight into potential mechanisms and pathways that Osx controls in osteoblasts and during the process of osteoblastic cell differentiation.

## Introduction

Bone formation is a developmental process involving the differentiation of mesenchymal stem cells to osteoblasts. It involves two processes: intramembranous and endochondral ossification. The vast majority of bones in the mammalian skeleton form by endochondral ossification which requires a cartilagenous template for osteoblast-directed mineral deposition. Fewer bones, particularly those in the skull, form by intramembranous ossification, a process in which mineralized bone forms directly from mesenchymal condensations. Osteoblast differentiation from mesenchymal stem cells occurs through a multi-step molecular pathway regulated by a complexity of transcription factors and signaling proteins, including Indian Hedgehog (Ihh), Runx2, Osterix (Osx), and Wnt signaling pathway proteins [1]. Ihh is required for endochondral ossification and is needed for the activation of Runx2 [2]. Both endochondral and intramembranous ossification require Runx2 which is involved in the differentiation of mesenchymal cells into preosteoblasts [3]. Osx is a downstream gene of Runx2. It is specifically expressed in osteoblasts and at low levels in prehypertrophic chondrocytes [4]. Osx is essential for the commitment of preosteoblastic cell differentiation into mature osteoblasts. Wnt signaling plays a role in bone differentiation and proliferation. The Wnt pathway is important for bone mass determination in the adult bone. This pathway affects different stages of bone formation and bone metabolism [5], [6].

Osx is an osteoblast-specific transcription factor required for bone formation and osteoblast differentiation. Osx knock-out mice completely lack mineralized bone while cartilagenous tissue is essentially normal [4]. Osx-null mouse embryos do not express osteoblast differentiation markers, such as osteocalcin (OC), alkaline phosphatase (ALP), and others. Osx is reported to inhibit the Wnt signaling pathway, suggesting a possible mechanism through which Osx inhibits osteoblast proliferation [7]. It is suggested that Osx coordinates both osteoblast differentiation and osteoblast proliferation during bone formation. Our recent observation that Osx inhibits the Wnt signaling pathway highlights the potential for novel feedback control mechanisms involved in bone formation.

The vitamin D receptor (VDR) is the nuclear hormone receptor of the vitamin D endocrine system whose principal role is to maintain calcium and phosphate homeostasis. The bioactive, hormonal ligand for VDR is 1,25-dihydroxyvitamin D_3_ [1,25-(OH)_2_D_3_]. Thus, VDR functions as a ligand-induced nuclear transcription factor to regulate the expression of genes in critical mineral-regulating target tissues such as the intestine, kidney, parathyroids, and bone in order to maintain appropriate mineral homeostasis. Both humans and mouse models that lack functional VDR display severe hypocalcemia, hypophosphatemia, rickets and osteomalacia highlighting a physiological significance of VDR in maintaining skeletal integrity [8], [9], [10]. Dietary calcium supplementation prevents the development of the undermineralized skeletal phenotype in humans with defective VDR and in VDR-null mice [11], [12] indicating that the role of VDR in bone is indirect or secondary to its role in maintaining appropriate intestinal and renal handling of calcium. Importantly, numerous studies also strongly support a direct role for VDR/1,25-(OH)_2_D_3_ in the osteoblast where one of its principal effects is to stimulate RANKL production and osteoblast-mediated osteoclastogenesis [13], [14]. Numerous bone-selective extracellular matrix proteins such as osteocalcin and osteopontin are VDR target genes [15], [16]. 1,25-(OH)_2_D_3_ is known to have pleotropic, direct effects on osteoblast differentiation, inhibiting differentiation at the early stages and promoting late stage osteoblast differentiation [17]. The expression levels of VDR may be key in this regard and are highlighted by the osteoblast-directed transgenic expression of VDR [18], [19]. These mice show an increase in cortical and trabecular bone suggesting an anabolic function under conditions of high order VDR expression in osteoblasts. Thus, an understanding of the systems and pathways governing VDR expression levels in osteoblasts may help uncover the basis for pleotropism of VDR/1,25-(OH)_2_D_3_ in osteoblast function.To identify the possible downstream targets of Osx, we performed quantitative real-time RT-PCR to compare RNA levels of different genes between wild type and *Osx* knock-out mice. In this current study, quantitative real-time RT-PCR results demonstrate that VDR expression is suppressed in the absence of Osx, and enhanced when Osx is overexpressed. This suggests that Osx may control VDR gene expression. Additional evidence from this study indicates that Osx targets the VDR gene promoter directly. This provides a new, additional mechanism through which Osx controls osteoblast activity.

## Methods

### Animals and Genotyping

Wild type and *Osx*-null mice are on a C57BL genetic background. All mice were bred and maintained in a specific pathogen-free facility. Mice were genotyped using genomic DNA isolated from the tails as previously described [4]. VDR-null mice were generously provided by M. Demay (Massachusetts General Hospital and Harvard Medical School, Boston, MA). This line of mice is on a homogeneous C57BL/6 genetic background. Mouse genotyping was performed with tail DNA as previously described [20]. All research protocols related to the VDR-null model were approved by the Institutional Animal Care and Use Committee of Case Western Reserve University, where this study was conducted. The animal protocol number is 2008-0017.

### RNA isolation and real-time RT-PCR

Total RNA was purified from the calvaria of E18.5 wild type and *Osx*-null mouse embryos with TRIzol reagent (Invitrogen) with additional purification using an RNeasy mini kit (Qiagen). Total RNA from C2C12 cells was isolated directly using an RNeasy Mini Kit according to the manufacturer's protocols (Qiagen). RNA was subjected to quantitative real-time RT-PCR, using the TaqMan One-Step RT-PCR Master Mix reagent (Applied Biosystems). Relative transcript levels were analyzed by real-time PCR in a 20 µl reaction volume on 96-well plates, using an ABI 7500 real-time PCR system (Applied Biosystem). Transcript levels were normalized to glyceraldehyde-3-phosphate dehydrogenase (GAPDH) levels. All reactions were done in duplicate and all experiments were repeated at least three times.

### Plasmid constructs and subcloning

Subcloning was performed as previously described with modifications [21]. Progressive deletion fragments of the *VDR* promoter region were generated by PCR using mouse genomic DNA as a template and subcloned into the XhoI and MluI sites of the pGL-3 vector. Primer sequences were designed based on the published sequence of the murine VDR promoter and transcriptional start site [22]. Primers were obtained from Integrated DNA Technologies (IDT) (Coralville, IA). The primer sequences were as follows: 1) VDR-Xho-3 5′GCG CCT CGA GAC AAG CAG AGA CTG CTC AGC AC, 2) VDR-Mlu-1K 5′ GCG CAC GCG TAC TAA CCG CCA GGC TGG GCT CTC ATC, 3) VDR-Mlu-500 5′GCG CAC GCG TCC TCC AAT CTG GTT TTC TTG G, 4) VDR-Mlu-250 5′GCG CAC GCG TAC GTG GAT TTG CAC ACA CGA C, 5) VDR-Mlu-120 5′GCG CAC GCG TGT CAA GAA AGT TTC AGG GCT TC. Point mutations were introduced in the VDR-120 promoter construct using the QuickChange site-directed mutagenesis kit (Stratagene) and the following primers: 1) VDR-M1-1 5′GCG GTC CGG GAA CAA AAC CTT GCG GGG GCG GGG CCA GGT GCT GAG, 2) VDR-M1-2 5′ CTC AGC ACC TGG CCC CGC CCC CGC AAG GTT TTG TTC CCG GAC CGC, 3) VDR-M2-1 5′GCG GTC CGG GGG CGG GGC CTT GCG GGA ACA AAA CCA GGT GCT GAG, 4) VDR-M2-2 5′ CTC AGC ACC TGG TTT TGT TCC CGC AAG GCC CCG CCC CCG GAC CGC. All deletion and mutant constructs were verified by DNA sequencing.

### Cell culture and transient transfection assay

HEK293 cells (ATCC) were cultured in high glucose Dulbecco's modified Eagle's medium (GIBCO) supplemented with 10% fetal bovine serum and 100 units/ml penicillin plus 100 µg/ml streptomycin at 95% air/5% CO_2_ humidified incubator. Cells were plated in 12-well plates, cultured to 60–80% confluence and transfected with FuGENE 6 (Roche) according to the manufacture's instruction. Cells were cotransfected with 300 ng of *VDR* promoter luciferase reporter, an Osx expression plasmid (pEX-Osx) as indicated, and 25 ng of pSV2-beta-gal. After transfection, cells were incubated for 24 h before harvest. The reporter assays were analyzed with a BD Monolight system (BD Biosciences). Luciferase activity was normalized to β-galactosidase activity. All transfection experiments were repeated at least three times. Values were presented as the mean ± standard deviation (S.D.). MC3T3 cells (ATCC) were cultured in Alpha Minimum Essential Medium with ribonucleosides, deoxyribonucleosides, 2 mM L-glutamine and 1 mM sodium pyruvate, but without ascorbic acid (GIBCO), and supplemented with 10% FBS and penicillin plus streptomycin. For MC3T3 osteoblast differentiation experiments, osteogenic factors were added into the medium, including BMP2 (300 ng/ml), ascorbic acid (50 µg/ml) and β–glycerophosphate (10 mM). This differentiation medium was changed every three days. Stable C2C12 mesenchymal cells expressing Osx were generated with the pTet-off Advanced Inducible Gene Expression System (Clontech) as previously described [7]. C2C12 cells were cultured in Dulbecco's modified Eagle's medium with the following additives to maintain selection and control Osx expression; G418 (200 µg/ml), hygromycine (150 µg/ml), and with or without doxycycline (Dox, 20 ng/ml). Osx expression was induced by the addition of media lacking doxycycline, a member of the tetracycline group of antibiotics.

### siRNA interference

MC3T3 cells were transfected with siRNA directed against mouse Osx and C2C12 cells were transfected with siRNA directed against mouse VDR using Lipofectamine 2000. siRNA oligos were purchased from Thermo Scientific Dharmacon, and siGENOME Lamin A/C Control siRNA was used as a non-specific control. Cells were cultured in 6-well plates. Cells were plated in 1 ml of growth medium without antibiotics 1 day prior to transfection and the cells were 30–50% confluent at the time of transfection. The final siRNA concentration was 100 nM and each well received 100 µl of the siRNA∶Lipofectamine. 2000 complex in Opti-MEM I medium.

### Protein purification and Western blot

Protein was isolated by acetone precipitation from the RNeasy cell lysates according to the manufacturer's protocol (QIAGEN). The protein pellet was dissolved in 1% SDS buffer, warmed for 15 min at 55°C, and centrifuged for 5 min at 14000 rpm. Protein concentrations in the supernatant were determined using a BCA Protein Assay Kit (Pierce). Proteins were separated on 10% SDS-PAGE gels and transferred to a PVDF membrane followed by Western blot analysis. Briefly, 3% milk in TBS containing 0.1% Tween-20 was used to block non-specific binding. The blot was subsequently incubated with an anti-VDR rabbit polyclonal antibody (1∶200, Abcam), an anti-ALP rabbit polyclonal antibody (1∶100, Santa Cruz Biotechnology), an anti-OC rabbit polyclonal antibody (1∶100, Santa Cruz Biotechnology) or an anti-Osx rabbit polyclonal antibody (1∶200, Santa Cruz Biotechnology) followed by a secondary antibody (peroxidase-conjugated anti-rabbit IgG 1∶5000, Sigma). After each antibody incubation, blots were extensively washed in TBS containing 0.1% Tween-20. For detection, the ECL kit (Amersham Life Sciences) was used according to the directions of the manufacturer.

### Chromatin immunoprecipitation (ChIP) assay

The Chromatin Immunoprecipitation (ChIP) Assay Kit was from Millipore. ChIP assays were performed as previously described [23] with some modifications. Briefly, calvarial cells were isolated from wild-type newborn mice, and were cultured in DMEM supplemented with 10% FBS. Formaldehyde was used to cross link the cells for 10 min, and crosslinking was quenched with glycine. Cells were harvested, rinsed with PBS, and cell pellets were resuspended in 1 ml of lysis buffer. After sonication, 100 µl of sheared chromatin was diluted to 1 ml with IP dilution buffer for each immunoprecipitation. The chromatin solution was pre-cleared with 60 µl of protein G Agarose beads at 4°C for 1 hr. The pre-cleared chromatin was collected and incubated at 4°C overnight with 5 µg of anti-Osx antibody or IgG as a negative control. Anti-Osx antibody was created by immunizing rabbits with a 14-amino acid peptide (AHGGSPEQSNLLEI) located at the C-terminus of the Osx polypeptide. The antiserum was affinity-purified over a 3 M Emphaze Biosupport Medium AB1 column (Pierce) coupled to the 14-amino acid peptide and was eluted. It was then dialyzed against Tris-buffered saline. The immune complexes were precipitated with 60 µl of protein G Agarose beads at 4°C for 1 hr. The antibody-protein-DNA immunocomplexes were washed and subsequently eluted twice with 100 µl of elution buffer. Formaldehyde cross-linking was reversed by heating at 65°C overnight with the addition of 5 M NaCl. All the samples were digested with RNase A and proteinase K. The DNA was purified using spin columns, and analyzed by real time PCR. The primer sets used for amplification of VDR promoter regions were obtained from IDT, and the sequences were as follows: Primer Set 1, VDR-1: 5′TAC TGT TCC ACG GAA GGC AGA C and VDR-2: 5′ ACA AGC AGA GAC TGC TCA GCA G; Primer Set 2, VDR-D-1: 5′ TGG CCG AGT TAC AGA ATT CC and VDR-D-2: 5′ CAT GTG CCA GAT CCT CTG TG. Data were normalized using GAPDH.

### Statistical Analysis

All experiments were repeated a minimum of 3 times. Data was reported as the mean ± standard deviation (S.D.). Comparisons were made between groups by Student's t test with p<0.05 being considered as statistically significant.

## Results

### VDR expression is reduced in the absence of Osx

**Figure 1:**
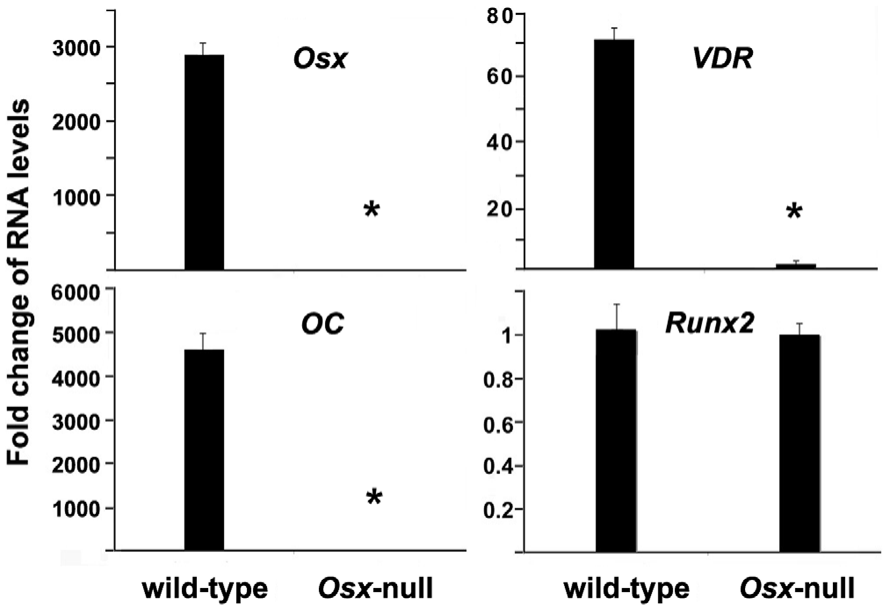
Osx ablation reduces VDR gene expression *in vivo*. Calvaria RNAs were isolated from E18.5 *Osx* wild-type and *Osx*-null embryos. RNA expression levels for Osx, osteocalcin (OC), Runx2 and VDR were analyzed by real-time RT-PCR. The level of each RNA from *Osx*-null calvaria was normalized to a value of 1. *: A star indicates statistical significance compared to Osx wild type group.

To identify possible downstream targets of Osx in the developing mouse skeleton, we performed quantitative real-time RT-PCR on RNA from wild type and *Osx* knockout mice in order to compare RNA levels of several genes. RNA was isolated from calvaria of E18.5 mouse embryos. As shown in Fig. 1, *Osx* expression was readily detected in wild-type calvaria and predictably absent in *Osx*-null calvaria. Likewise, osteocalcin expression, a marker of mature osteoblasts, was dramatically reduced in Osx-null calvaria compared to wild-type tissue. In contrast, Runx2, a gene that is upstream of Osx in the osteoblast differentiation pathway, was unchanged in the Osx-null calvaria. Interestingly, we observed that *VDR* expression was suppressed by greater than 70-fold in *Osx*-null calvaria compared with that in wild-type calvaria. The significant decrease in *VDR* RNA level in *Osx* knock-out mice suggests that *Osx* regulates *VDR* gene expression.

### Overexpression of Osx activates VDR gene expression

**Figure 2:**
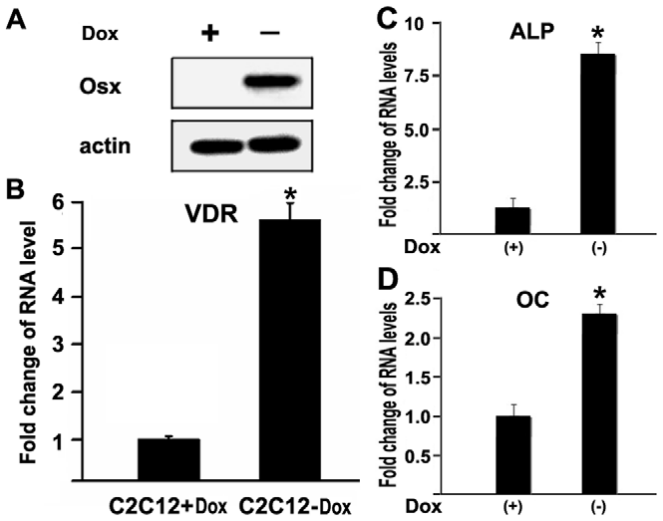
Overexpression of Osx activates VDR gene expression in C2C12 mesenchymal cells. (A) Western immunoblot analysis of the Dox-regulated Osx-expressing C2C12 cells. Osx expression is turned on in the absence of Dox. Beta-actin was used as a loading control. (B) VDR mRNA levels in a stable, Tet-off C2C12 mesenchymal cell line. RNA was obtained from cultures treated with or without Doxycycline. Osx expression is induced in the absence of Doxycycline in this line. VDR mRNA levels were quantitated by real-time RT-PCR. The VDR RNA level obtained from the cells cultured with Dox was normalized to a value of 1. Values are presented as the mean ±S.D. (C) RNA expression level for the osteoblastic marker gene alkaline phosphatase (ALP). (D) RNA expression level for osteoblastic gene osteocalcin (OC). Conditions are identical to those described in panel B. A paired *t*-test was performed comparing Dox (−) and Dox (+) groups. *: A star indicates statistical significance compared to Dox (+) group.

To test whether forced expression of Osx stimulated VDR gene expression, we used a stable, Osx-inducible C2C12 mesenchymal cell line that was described previously [7]. In this line, expression of Osx is dramatically induced upon removal of doxycycline (Dox)(Fig. 2A). Total RNA was purified from this line following culture in the presence or absence of Dox and *VDR* expression was quantitated by real-time RT-PCR. As shown in Fig. 2B, in the absence of Dox (i.e., overexpression of Osx), *VDR* expression was enhanced 5.5 fold. In this cell system, ectopic Osx expression also coincides with the expression of osteoblast marker genes ALP (Fig. 2C) and osteocalcin (OC; Fig. 2D). These data indicate that increased expression of Osx results in increased expression of VDR and osteoblastic marker genes in the C2C12 system.

### Inhibition of Osx by siRNA reduces VDR gene expression in osteoblasts

**Figure 3:**
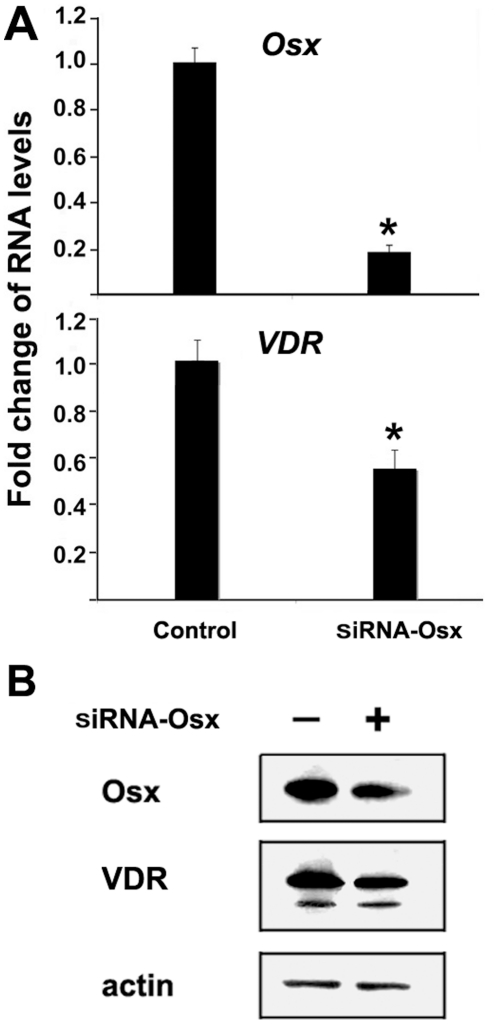
SiRNA-directed knockdown of Osx impairs VDR gene expression in MC3T3 osteoblasts. (A) RNA expression levels as determined by quantitative real-time RT-PCR. MC3T3 osteoblasts were transfected with siRNA targeting mouse Osx. RNA was isolated 24 h post-transfection and quantitated by real-time RT-PCR. The RNA level from the control siRNA group was normalized to a value of 1. Values were presented as the mean ±S.D. A paired *t*-test was performed comparing si-control group and si-Osx group. (B) Western analysis of the Osx knockdown. Protein was isolated by acetone precipitation of whole cell lysates and then analyzed by western blot using rabbit anti-VDR or anti-Osx polyclonal antibodies. Beta-actin was used as a loading control.

To establish a direct effect of Osx on VDR expression, we used siRNA to knockdown Osx expression in MC3T3 osteoblast cells. MC3T3 cells were chosen for this approach because they express higher, more readily detected levels of Osx and VDR. Real-time RT-PCR and western-blots were performed to analyze mRNA and protein expression levels, respectively. As shown in Fig. 3A, when Osx RNA expression was decreased by 80% using siRNA targeted against Osx, VDR RNA levels were reduced by approximately 42%. The decrease in VDR expression mediated by Osx-knockdown was also evident at the protein level as depicted in Fig. 3B. Therefore, these data support a role for Osx in enhancing VDR gene expression in osteoblasts.

### Osx gene expression is independent VDR *in vivo*

**Figure 4:**
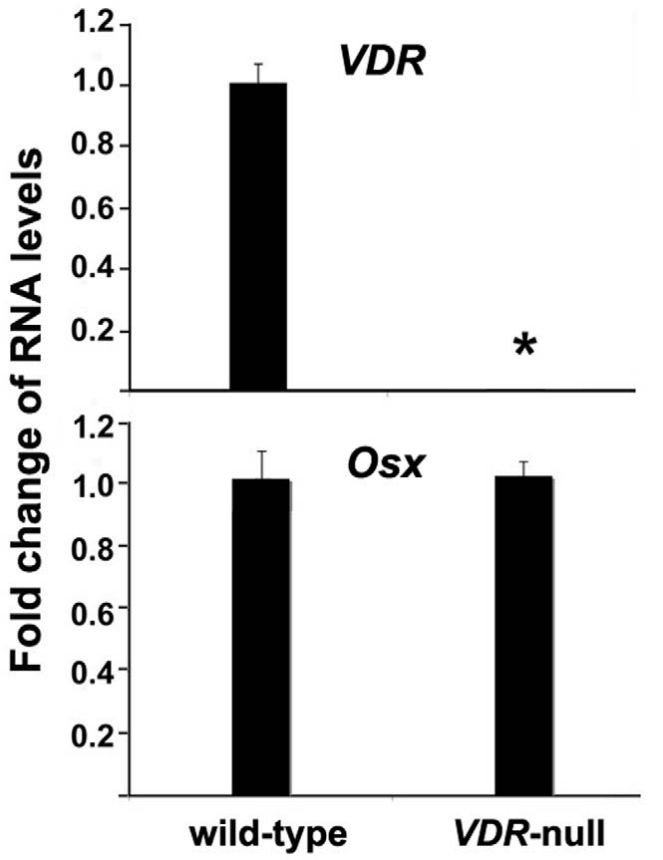
VDR ablation has no effect on Osx gene expression *in vivo*. Calvarial RNAs were isolated from E18.5 wild-type or *VDR*-null embryos. RNA levels for VDR and Osx were analyzed by real-time RT-PCR. The level of each RNA from wild type calvaria was normalized to a value of 1 shown in the mean±S.D. A paired *t*-test was performed comparing wild-type and VDR-null group.

To test whether a complementary relationship exists, that is, whether Osx is affected by VDR status in vivo, we examined Osx gene expression levels in wild-type and VDR-null E18.5 calvaria. As shown in Fig. 4, VDR expression was undetectable in *VDR*-null calvaria as expected. Osx expression levels remained unchanged in the absence of VDR. These data support the concept that Osx is an upstream gene of VDR.

### Osx activates the murine VDR promoter through GC-rich regions in the immediate 5′ region

**Figure 5:**
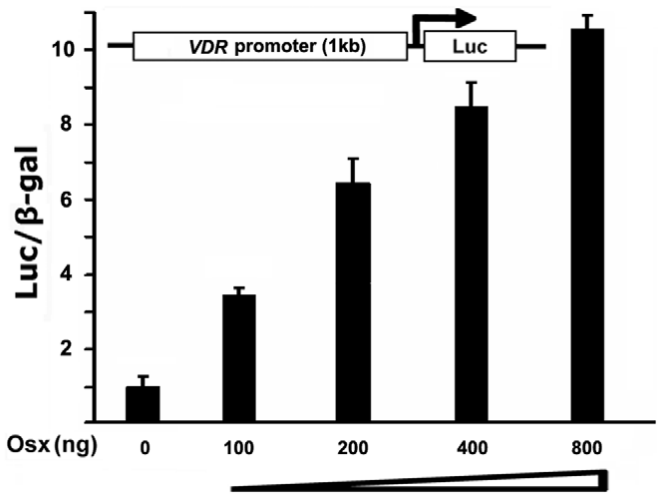
Osx activates the *VDR* promoter in a dose-dependent manner. HEK293 cells were transfected with a 1 kb *VDR* promoter-luciferase reporter gene without or with increasing amounts of an Osx-expression plasmid as indicated. Luciferase activity was normalized by β-galactosidase activity. Values are presented as the mean ±S.D.

**Figure 6:**
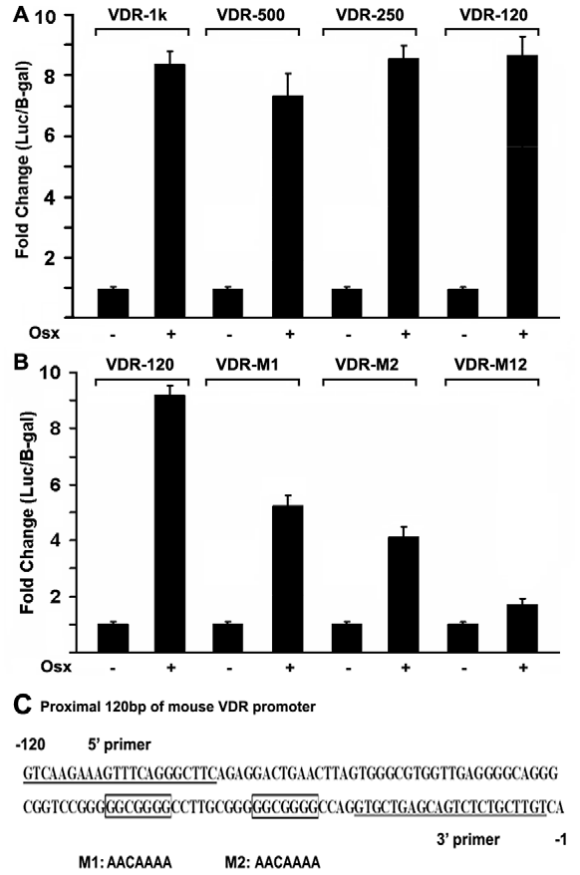
Identification of the Osx binding site in the promoter of *VDR* gene. (A) Deletion analysis of the *VDR* promoter-reporter construct. VDR-1 kb, VDR-500 bp, VDR-250 bp and VDR-120 bp promoter-reporter plasmids (300 ng each) were cotransfected with 400 ng of the Osx expression plasmid in HEK293 cells. Twenty-four hours post-transfection, cell extracts were prepared and analyzed for luciferase activity and normalized to β-galactosidase activity. (B) Two GC-rich elements in VDR-120 are responsible for *VDR* promoter reporter activation by Osx. The promoter mutants VDR-M1, VDR-M2 and VDR-M12 were transfected into HEK293 cells and analyzed as described in panel A. Luciferase activity was normalized by β-galactosidase activity. (C) A diagram of the proximal 120 bp region of the mouse VDR promoter. A 5′ primer and 3′ primer were used to subclone the VDR-120 bp promoter reporter plasmid. M1 refers to point mutations of VDR-M1, and M2 refers to point mutations of VDR-M2. VDR-M12 in (B) contains both M1 and M2.

Results from primary mouse calvarial cells, the stable C2C12 mesenchymal cell line and the MC3T3 osteoblastic cell line strongly indicate that Osx upregulates VDR gene expression. To test whether this is a direct effect of Osx on the VDR promoter, we generated a luciferase reporter construct driven by 1 kb of 5′-flanking sequence from the native, murine *VDR* promoter. This construct was cotransfected with different amounts of an Osx expression plasmid (pEX-Osx) into HEK293 cells. As illustrated in Fig. 5, increasing amounts of the Osx expression plasmid resulted in increased levels of VDR promoter-reporter activity in this cell system. To define the precise Osx regulatory region in the VDR promoter, we generated a series deletion constructs of the *VDR* promoter reporter and tested them in our HEK293 transfection assay. As shown in Fig. 6A, Osx activated all of the *VDR* promoter reporters similarly, including the VDR-1 kb, VDR-500 bp, VDR-250 bp and VDR-120 bp constructs. These data indicate that the putative regulatory or binding elements for Osx reside within the VDR 120 bp promoter region. Previous studies show that Osx belongs to the Sp/XKLF family of transcription factors that bind to GC-rich sequences of target gene promoters to regulate gene expression [4]. Sequence analysis of the active 120 bp region of *VDR* promoter reveal two potential, GC-rich binding sites for Osx located at positions −51–−45 and −35–−29, relative to the transcriptional start site. To study which binding site is responsible for *VDR* promoter activation by Osx, we disrupted each element by site-directed mutagenesis. The two mutants were designated VDR-M1 and VDR-M2 in which the guanines (Gs) in each element were replaced with adenines (As) (Fig. 6C). As shown in Fig. 6B, Osx activation of the M1 and M2 VDR promoter mutants was only 42% and 53% respectively, that of the wild-type VDR-120 bp promoter. Finally, we generated a double mutant in which both GC-rich elements were disrupted (VDR-M12). As shown in Fig. 6B, Osx expression only modestly activated this double mutation (<2-fold). Cumulatively, these data revealed that the two GC-rich sequences residing between −51 and −29 relative to the transcriptional start site, function as Osx-responsive elements that mediate the activation of the VDR promoter by Osx.

### Endogenous Osx associates with the native VDR promoter *in vivo*

**Figure 7:**
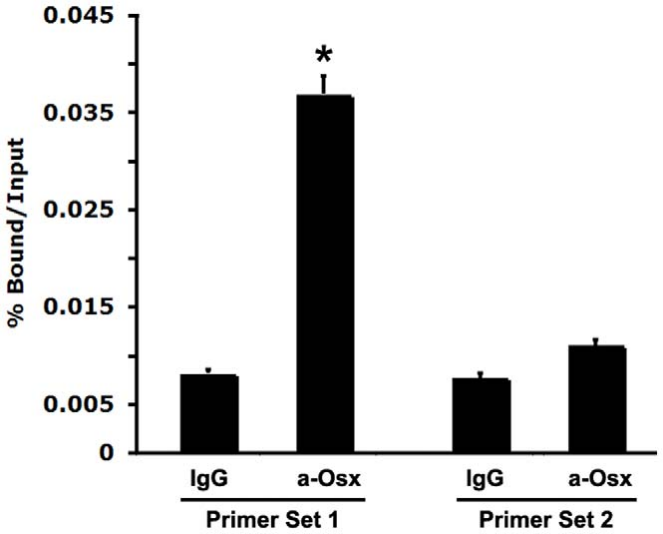
Endogenous Osx in primary osteoblasts is associated with the native *VDR* promoter *in vivo*. Chromatin Immunoprecipitation (ChIP) assays were conducted using primary calvarial osteoblasts isolated from new born wild-type mice. Anti-Osx antibody (a-Osx) was used for ChIP analysis, and IgG was used as a negative control. The precipitated chromatin was analyzed by quantitative real-time PCR. As described in the Methods, primer Set 1 corresponds to a segment covering two GC-rich elements within 120 bp the *VDR* promoter. As a negative control, Primer Set 2 covers a distal 3 kb region of the *VDR* promoter, which does not contain GC-rich sequences. A paired *t*-test was performed comparing IgG and a-Osx group.

The studies above indicate that Osx can positively regulate VDR expression and activate the *VDR* promoter *in vitro* through two GC-rich sequences in *VDR* promoter. However, it is currently unknown whether endogenous Osx associates with the native *VDR* promoter *in vivo*. To address this question, chromatin immunoprecipitation (ChIP) assays were carried out to examine whether Osx could bind to native *VDR* promoter in primary calvarial osteoblasts isolated from new born wild-type mice. Crosslinked extracts were immunoprecipitated with antibodies against Osx or control IgG. Following reversal of the crosslinks, DNA was recovered and analyzed by quantitative real time PCR using primers designed to amplify the Osx-responsive region covering the two GC-rich sequences of VDR-120 bp promoter (Primer Set 1) or a distal upstream 3 kb, non-responsive region (Primer Set 2) as a control to demonstrate response element selectivity. Fig. 7 demonstrated that Osx was associated with the *VDR* promoter region containing the two GC-rich sequences (Primer Set 1) compared with IgG control group. However, Osx was not associated with the *VDR* distal 3 kb promoter region lacking a GC-rich sequence (Primer Set 2), demonstrating that the observed Osx-DNA association was specific. Thus, these data indicate that endogenous Osx associates with the native *VDR* promoter in primary osteoblasts *in vivo*.

### VDR is involved in Osx-induced osteoblast differentiation

**Figure 8:**
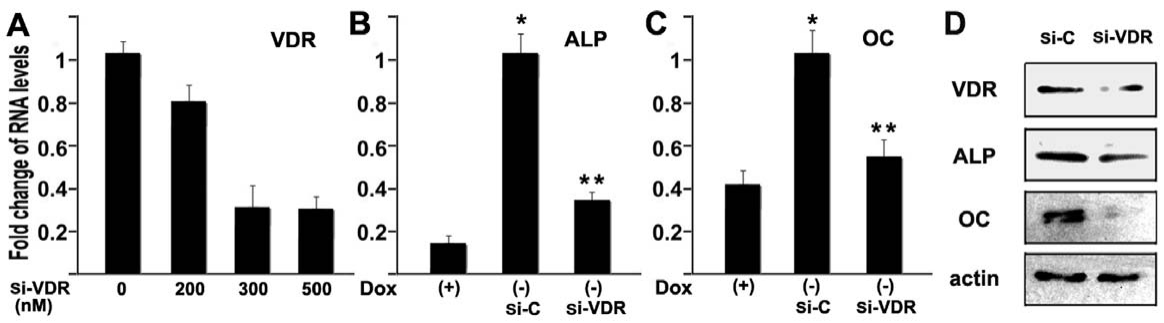
Osx-regulated expression of VDR is important in Osx-induced osteoblast differentiation. (A) SiRNA directed against VDR knocks down VDR expression. C2C12 mesenchymal cells stably expressing Osx were transfected with VDR siRNA at different concentrations. RNA was isolated and specific RNA levels were measured by real-time RT-PCR. (B,C) Cells were transfected with 300 nM of siRNA targeting VDR, and Osx expression was induced by removing Dox. Osteoblast marker gene expression was examined including (B) ALP and (C)osteocalcin (OC). The RNA level from control siRNA group (Si-C) was normalized to a value of 1. Values were presented as the mean ±S.D. *: A star indicates statistical significance compared to Dox (+) group (p<0.05). **: Two stars indicate statistical significance compared to si-control (Si-C) (p<0.05). (D) Western analysis of the VDR knockdown. C2C12 stable cells without Dox were transfected with 300 nM of siRNA targeting VDR. Protein was isolated from whole cell lysates of control siRNA group (si-C) and VDR siRNA group (si-VDR) and then analyzed by western blot using rabbit anti-VDR, anti-ALP or anti-OC polyclonal antibodies. Beta-actin was used as a loading control.

To determine if VDR gene regulation by Osx is important for appropriate control of osteoblastic gene expression, we tested the effects of VDR siRNA on Osx-induced osteoblast marker gene expression. Using our Tet-off C2C12 mesenchymal cell line, we observed that forced expression of Osx dramatically induced osteoblast marker gene expression, including ALP and osteocalcin (Fig. 8). siRNA against VDR reduced VDR expression significantly (66% at 300 nM, Fig. 8A). Importantly, VDR siRNA significantly inhibited Osx-induced expression of ALP and OC (Fig. 8B and C). The decreases in ALP and OC expression mediated by VDR-knockdown were also evident at the protein level as shown in Fig. 8D. These data suggest that the regulated expression of VDR by Osx is important for appropriate expression of osteoblastic marker genes. They further support our hypothesis that VDR is an important downstream target gene of Osx, and may participate in the regulation of critical genes involved in osteoblast differentiation and development.

**Figure 9:**
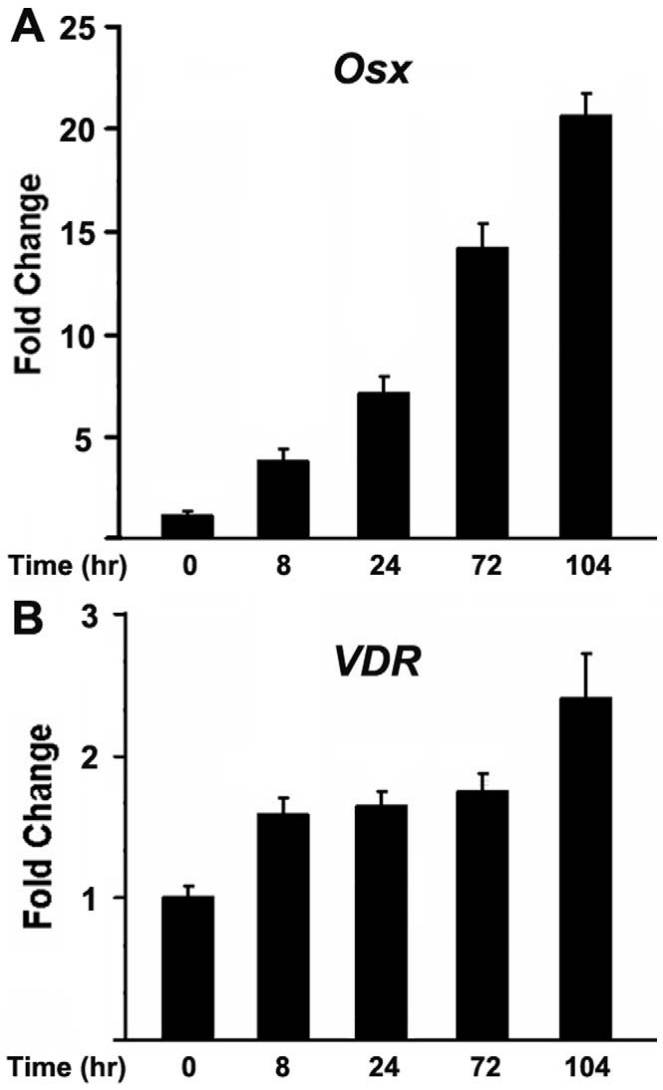
Both Osx and VDR are upregulated during osteoblast differentiation. MC3T3 osteoblast differentiation experiments were performed in which osteogenic factors were added into the medium, including BMP2, ascorbic acid and β–glycerophosphate. MC3T3 osteoblastic cells were harvested after 0 hr, 8 hr, 24 hr, 72 hr and 104 hr after incubating with differentiation medium. RNA was isolated from cell lysates. RNA levels for VDR and Osx were analyzed by real-time RT-PCR. The level of RNA from 0 hr was normalized to a value of 1. Values were presented as the mean ±S.D.

To examine the coordinate expression of Osx and VDR during osteoblast differentiation, preosteoblastic MC3T3-E1 cells were cultured in osteogenic media containing BMP2, ascorbic acid and β–glycerophosphate. Cells were harvested at various times following the addition of osteogeneic media. RNA was isolated from cell lysates, and analyzed by realtime RT-PCR. As shown in Fig. 9, both Osx and VDR expression were significantly enhanced throughout this early phase of osteoblast differentiation at times when early osteoblastic markers such as alkaline phosphatase increased (data not shown). These data show that both Osx and VDR are coordinately upregulated during osteoblast differentiation.

## Discussion

Osx is an osteoblast-specific transcription factor that controls the expression of essential genes needed for appropriate osteoblast differentiation and function. Despite the discovery of its significance in skeletal physiology a decade ago [4], relatively little is known about direct target genes for Osx and molecular mechanisms through which Osx regulates transcription. The findings presented here provide important, new insights into these two general, poorly understood areas of Osx biology.

First, we identified VDR as an Osx target gene. This is supported by the Osx-directed gene expression studies showing coordinate expression of Osx and VDR in several *in vivo* and *in vitro* model systems. For example, Osx-null calvaria displayed defective *in vivo* expression of VDR compared to wild-type calvaria in mice. Primary osteoblast cultures obtained from wild-type calvaria also had markedly impaired VDR gene expression when Osx expression was knocked-down using siRNA targeting strategies (Fig. 3) and a Tet-off inducible cell system revealed that ectopic expression of Osx resulted in an increase in VDR transcript level (Fig. 2B). Importantly, a direct regulation of VDR gene transcription by Osx was evident in the ability of recombinant Osx to activate VDR promoter-reporter constructs, thus indicating that the RNA expression studies were likely due to the effects of Osx expression on VDR gene transcription. The broader implication of these studies is that the VDR gene can now be added to a small, but growing list of osteoblastogenic factors and pathways that are regulated by Osx in the osteoblast [7], [24], [25].

In terms of mechanism, Osx is an SP/KLF family member that presumably functions by binding directly to DNA promoter elements via an SP1-like DNA-binding domain consisting of three C2H2-type zinc fingers located within its C-terminus [4]. While consensus Osx-binding DNA elements have not been rigorously defined as yet, they likely are GC-rich in nature based on the similarities of DNA-binding domains and elements among this family of transcription factors. We previously reported on similar elements controlling Osx-activation of the DKK1 and sclerostin promoters [7], [24]. Indeed, two such GC-rich regions exist in the most proximal regions of the murine VDR promoter residing immediately adjacent to its transcriptional start site [22]. Importantly, our studies define these two elements as critical for mediating the transcriptional activation of the VDR promoter by Osx. Mutation of each element (M1 or M2) reduced Osx-directed activation of the VDR promoter by approximately 50% and mutation of both elements nearly abolished the response (Fig. 6B). Of course, limitations of this particular approach include the heterologous expression and somewhat artificial nature of the plasmid DNA constructs used as a measure of a biologically relevant transcriptional event. Thus, the chromatin immunoprecipitation approaches (Fig. 7) strongly support the reporter gene expression data. These studies clearly demonstrated that endogenous Osx was associated with the GC-rich proximal region of the native VDR promoter in primary osteoblastic cells. These studies help establish Osx mechanisms involving direct binding of Osx to sequence specific, GC-rich promoter elements to activate the expression of genes in osteoblastic cells.

Osx is considered as a master regulator of osteoblast differentiation [4], [7]. Unfortunately, the cellular and molecular mechanisms governing Osx control of osteoblast differentiation and function are still not well characterized. Osx is known to function downstream of Runx2 during osteoblast differentiation [4], [26] and Osx is required for the expression of osteoblast-specific markers, such as type I collagen, osteocalcin, bone sialoprotein, osteonectin and osteopontin [4]. Interestingly, several of these genes are also regulated by VDR and 1,25-(OH)_2_D_3_ in the osteoblast [15], [16]. This fact, combined with our observation that VDR is an Osx target gene, strongly suggests that potential interplays between Osx and VDR in osteoblast gene expression, differentiation and/or function need to be explored in more detail in the future. Clearly, 1,25-(OH)_2_D_3_ and VDR have complex, seemingly disparate, roles depending on early versus late stages of osteoblast differentiation [17]. In early or preosteoblastic stages, 1,25-(OH)_2_D_3_ treatment suppresses type I collagen and alkaline phosphatase mRNA levels, while both genes are elevated by 1,25-(OH)_2_D_3_ in late-stage osteoblast differentiation. VDR and 1,25-(OH)_2_D_3_ directly inhibit the differentiation and function of early stage osteoblasts [27], [28], but enhances the activity of terminally differentiated osteoblasts [19], [29]. While its precise role is unresolved, our studies clearly indicate that Osx-directed regulation of VDR expression is a biologically relevant process that is needed for appropriate expression of osteoblastic cell marker genes. This is evident from the siRNA-mediated knockdown of VDR protein levels in the C2C12 model system (Fig. 8). Here, interfering with Osx-mediated induction of VDR gene expression interfered with the appropriate expression of alkaline phosphatase and osteocalcin, two of the most relevant biological markers of osteoblasts. Thus, while additional studies need to address other cell systems and the ultimate effects of Osx and VDR regulation on the full osteoblastic cell differentiation pathway progressing to the stage of mineralized matrix production, these early stage studies provide a sound biological context and relevance for Osx activation of VDR gene expression in this model system.

Most current therapeutic strategies for low bone density disorders such as osteoporosis focus on suppressing bone resorption. However, the main limitation of this approach is that the rate of bone loss is slowed, but not reversed. Thus, anabolic strategies targeting osteoblastic pathways and the stimulation of bone formation are highly desirable. Continued progress on Osx and downstream targets/pathways under its control will help determine the relevance of this system as an anabolic therapeutic targeting strategy to combat osteoporosis and other bone related diseases.
